# B‐cell lymphoma‐3 controls mesenchymal stem cell commitment and senescence during skeletal aging

**DOI:** 10.1002/ctm2.955

**Published:** 2022-07-08

**Authors:** Fuxiao Wang, Jiawei Guo, Sicheng Wang, Yili Wang, Jiao Chen, Yan Hu, Hao Zhang, Ke Xu, Yan Wei, Liehu Cao, Xiao Chen, Yingying Jing, Jiacan Su

**Affiliations:** ^1^ Institute of Translational Medicine Shanghai University Shanghai China; ^2^ Department of Orthopedics Trauma, Shanghai Changhai Hospital Naval Medical University Shanghai China; ^3^ Department of Orthopedics Shanghai Zhongye Hospital Shanghai China; ^4^ Department of Orthopedics Shanghai Baoshan Luodian Hospital Baoshan District Shanghai China

Dear Editor,

Skeletal aging is characterized by progressive bone loss and increased marrow adiposity.^1^, ^2^ Age‐related bone loss impairs the exercise activity of patients and increases the risk of fracture. Bone marrow mesenchymal stem cells (BMSCs) could differentiate into osteoblasts and adipocytes.^3^, ^4^ The senescence and differentiation shift of BMSCs plays a critical role in skeletal aging and osteoporosis. In our study, we demonstrated that B‐cell lymphoma 3 (Bcl‐3), a member of the inhibitor of κB (IκB),^5^, ^6^ attenuated BMSCs senescence and regulated BMSCs differentiation fate through manipulating Wnt signalling.^7^, ^8^

First, we showed that the level of Bcl‐3 from 1 to 18 months in mice was significantly decreased with aging (Figure 1A). Then, we generated Bcl‐3^–/–^ mice which displayed slightly smaller size than that of wild‐type (WT) mice and results of WB and qRT‐PCR confirm that Bcl‐3 was deleted in Bcl‐3^–/–^ mice (Figure S1A–C). Micro‐computed tomography (μCT) analysis (Figure 1B,C and Figure S1D) and H&E staining (Figure S1E) showed a significant bone loss in 10‐week‐old Bcl‐3^–/–^ mice compared with control. Bcl‐3 knockout also significantly increased bone loss following ovariectomy (OVX) by μCT (Figure 1D and Figure S1F). Calcein double labelling verified Bcl‐3^–/–^ mice had less endosteal, periosteal and trabecular bone formation than that of the WT counterparts, mineral apposition and bone formation was lower in Bcl‐3^–/–^ group (Figure 1E,F). In addition, the number of trabecular OCN‐positive cells in Bcl‐3^–/–^ mice was less than that in WT mice, while the number of FABP4‐positive adipocytes and trap‐positive cells were increased in Bcl‐3^–/–^ mice (Figure 1G–I and Figure S1G).

**FIGURE 1:**
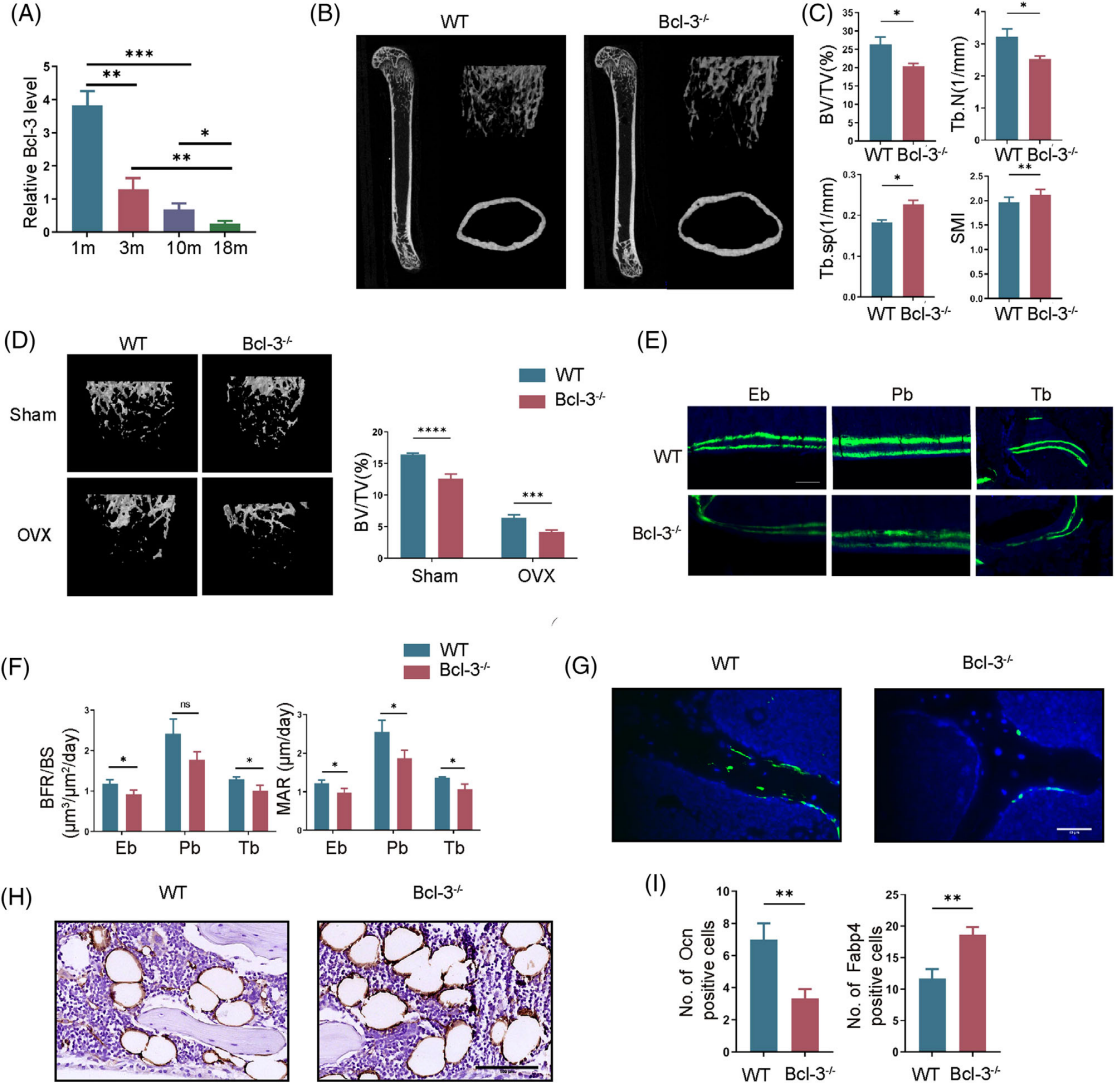
Deletion of Bcl‐3 exacerbates bone loss and reduces bone formation. (A) Bcl‐3 mRNA in femurs from 1 m, 3 m, 10 m and 18 m wild‐type (WT) male mice was assessed by qRT‐PCR. (*N* = 3 independent experiments). (B) Representative micro‐computed tomography (μCT_ images of femurs from 10‐week‐old Bcl‐3^–/–^ and WT male mice. (*N* = 3 mice/group). (C) Quantitative measurements of BV/TV, Tb.N, Tb.Sp and SMI in 10‐week‐old Bcl‐3^–/–^ and WT mice by μCT. (*N* = 3 mice/group). (D) Representative μCT images of femurs from 10‐week‐old Bcl‐3^–/–^ and WT female mice following sham and ovariectomy (OVX). (*N* = 3 mice/group). (E,F) Representative images of calcein double labelling of endosteal bone (Eb), periosteal bone (Pb) and trabecular bone (Tb) with quantification of BFR/BS and MAR in the femurs of 10‐week‐old WT and Bcl‐3^–/–^ male mice. (G) Representative immunostaining of OCN in 10‐week‐old Bcl‐3^–/–^ and WT male mouse femurs. Scale bar, 50 μm. (*N* = 3 mice/group). (H) Representative immunostaining of FABP4 in 6‐month‐old Bcl‐3^–/–^ and WT male mouse femurs. Scale bar, 100 μm. (*N* = 3 mice/group). (I) Quantification of immunostaining of OCN and FABP4. The data are presented as the mean ± SD. *P <.05; **P <.01; ***P <.005; ****P <.0001 vs. control group. Statistical analysis was performed using Student's *t*‐test (A, C, F and I) and two‐way ANOVA test (D). Primary antibodies: OCN, Abcam(ab93876); FABP4, Abcam(ab92501)

Then, we examined the differentiation potential, namely osteogenesis and adipogenesis of BMSCs after Bcl‐3 was silenced in vitro. BMSCs were isolated from 5‐week‐mice and identified by flow cytometry (Figure S2A,B). The osteogenesis of BMSCs was decreased when Bcl‐3 was knockdown verified by staining of alkaline phosphatase (ALP) and calcium nodules stained by Alizarin Red S (ARS) and the decreased mRNA levels of *Ocn*, *Osterix* and *Runx2* (Figure 2A,B). On the contrary, adipogenesis was increased in Bcl‐3‐knockdown BMSCs validated by oil red staining and the mRNA levels of *Fabp4, Adipoq* and *Pparγ* (Figure 2C,D). When Bcl‐3 was knocked down in BMSCs, the expression of FABP4 was increased, accompanied by decreased OSTERIX expression (Figure 2E). On the other hand, we found that Bcl‐3 overexpression could promote osteoblastic differentiation and inhibit adipogenic differentiation in senescent BMSCs treated by H_2_O_2_ (Figure S3A–F).

**FIGURE 2:**
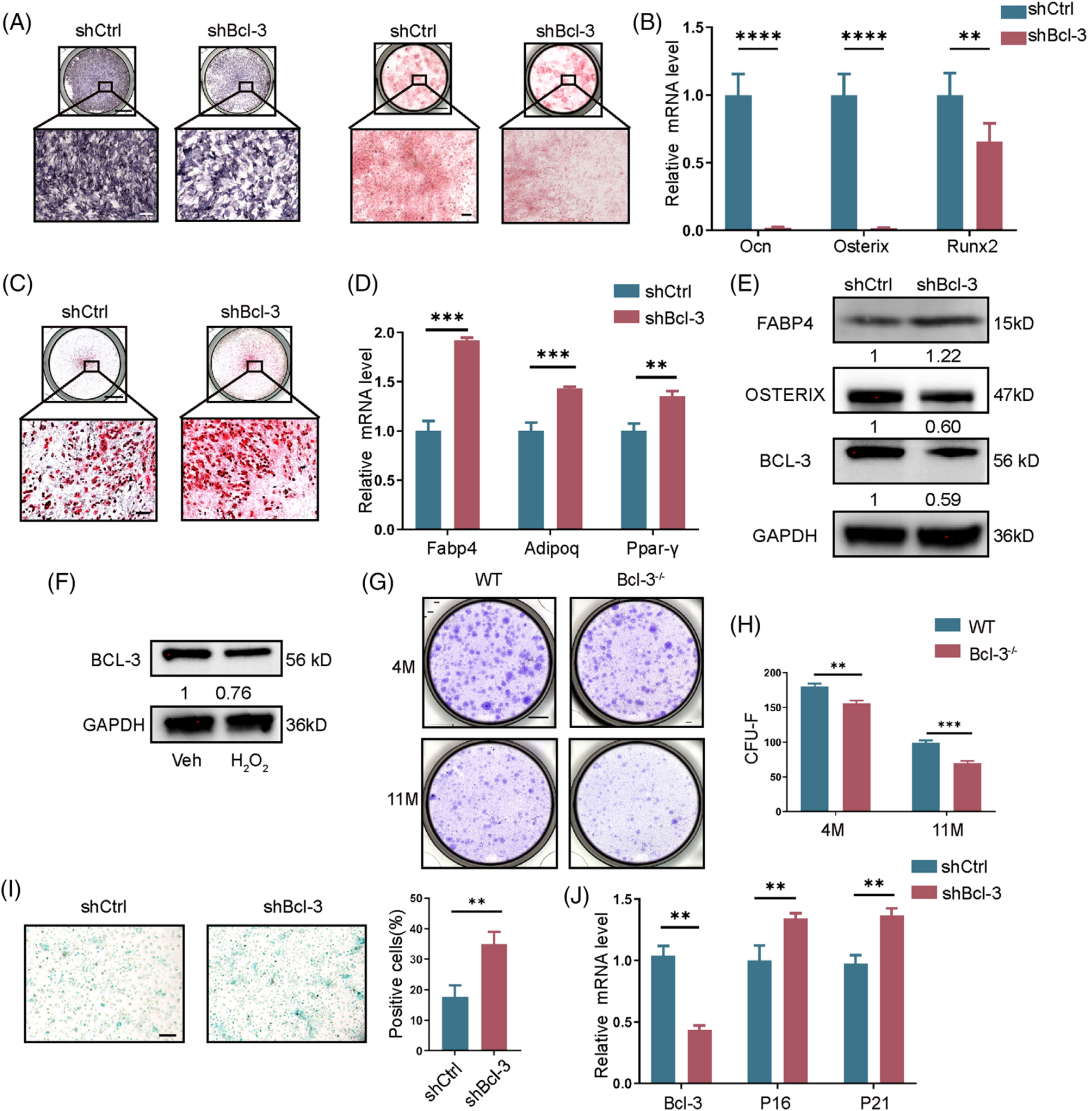
Bcl‐3 regulates adipo‐osteogenic differentiation of bone marrow mesenchymal stem cells (BMSCs) in vitro and protects BMSCs from senescence. (A) Bcl‐3 was knockdown in BMSCs and cultured in differentiation medium. Cell differentiation was assessed by alkaline phosphatase (ALP) and Alizarin Red S (ARS) staining after14 days of osteogenic induction. Scale bar, 5 mm (above) and 200 μm (below). (B) The expression of osteogenesis markers (OCN, Osterix and Runx2) were analysed 14 days after osteogenic induction of Bcl‐3 knock‐out BMSCs. (*N* = 4 independent experiments). (C) Bcl‐3 was knockdown in BMSCs and cultured in differentiation medium. Cell differentiation was assessed by oil red staining after 21 days of adipogenic induction. Scale bar, 5 mm (above) and 200 μm (below). (D) The expression of adipogenic markers (Fabp4, Adipoq and PPARγ) was analysed 6 days after adipogenic induction of Bcl‐3 knock‐out BMSCs. (*N* = 4 independent experiments). (E) Western blot detection of GAPDH, Bcl‐3, Osterix and Fabp4 protein in BMSCs. The data are presented as the mean ± SD. (F) Western blot detection of GAPDH and Bcl‐3 protein levels in H_2_O_2_ treated BMSCs. (G,H) Representative images and quantification of CFU‐Fs formed by cells from Bcl‐3^–/–^ and wild‐type (WT) mice. Scale bar, 0.5 mm. (*N* = 3 independent experiments). (I) SA‐β‐gal staining of Bcl‐3 knockdown BMSCs and quantitative analysis of SA‐β‐gal staining. Scale bar, 200 μm. (*N* =3 independent experiments). (J) Bcl‐3, P16 and P21 mRNA in Bcl‐3 knockdown BMSCs was assessed by qRT‐PCR. (*N* = 4 independent experiments). The data are presented as the mean ± SD. *P <.05; **P <.01; ***P < .005; ****P < .0001 vs. control group. Statistical analysis was performed using Student's *t*‐test (B, D, I and J) and two‐way ANOVA test (H). Primary antibodies: GAPDH, Abcam(ab181602); BCL‐3, Abcam(ab259832); FABP4, Abcam(ab92501); OSTERIX, Abcam(ab209484)

In aging BMSCs, the expression level of Bcl‐3 was decreased (Figure 2F and Figure S4A). For self‐renewable capacities, BMSCs of Bcl‐3^–/–^ mice showed fewer colony‐forming unit‐fibroblasts (CFU‐Fs) (Figure 2G,H). The number of SA‐β‐gal‐positive blue cells in Bcl‐3 knockdown BMSCs was significantly increased than control BMSCs (Figure 2I). The expression of aging makers, *P21* and *P16*, was significantly upregulated when Bcl‐3 was knockdown in BMSCs (Figure 2J). Moreover, Bcl‐3 overexpression reduced senescent cells numbers and the expression of aging makers after treated with H_2_O_2_ (Figure S4B,C). To sum up, Bcl‐3 was decreased during BMSCs senescence, and the supply of Bcl‐3 could protect BMSCs from aging.

To explore the molecular mechanisms, we performed RNA sequencing in shBcl‐3 and shCtrl BMSCs. The results of gene ontology (GO) revealed that the downregulated genes following Bcl‐3 knockdown were mainly enriched in ossification, bone mineralization, bone growth, etc. Kyoto encyclopaedia of genes and genomes (KEGG) revealed that Bcl‐3‐knockdown BMSCs influenced Wnt/β‐catenin signalling pathways (Figure 3A,B). Heat map showed that relative expression levels of genes in the Wnt pathway were decreased in shBcl‐3 BMSCs compared to shCtrl BMSCs (Figure 3C). In addition, we found that Wnt/β‐catenin signalling was downregulated in Bcl‐3 knockdown BMSCs indicated by gene set enrichment analysis (GSEA, Figure 3D). The expression levels of genes in Wnt signalling were decreased when Bcl‐3 was knockdown in BMSCs (Figure S5A,B) and increased in Bcl‐3‐overexpressed BMSCs (Figure S5C,D).

**FIGURE 3:**
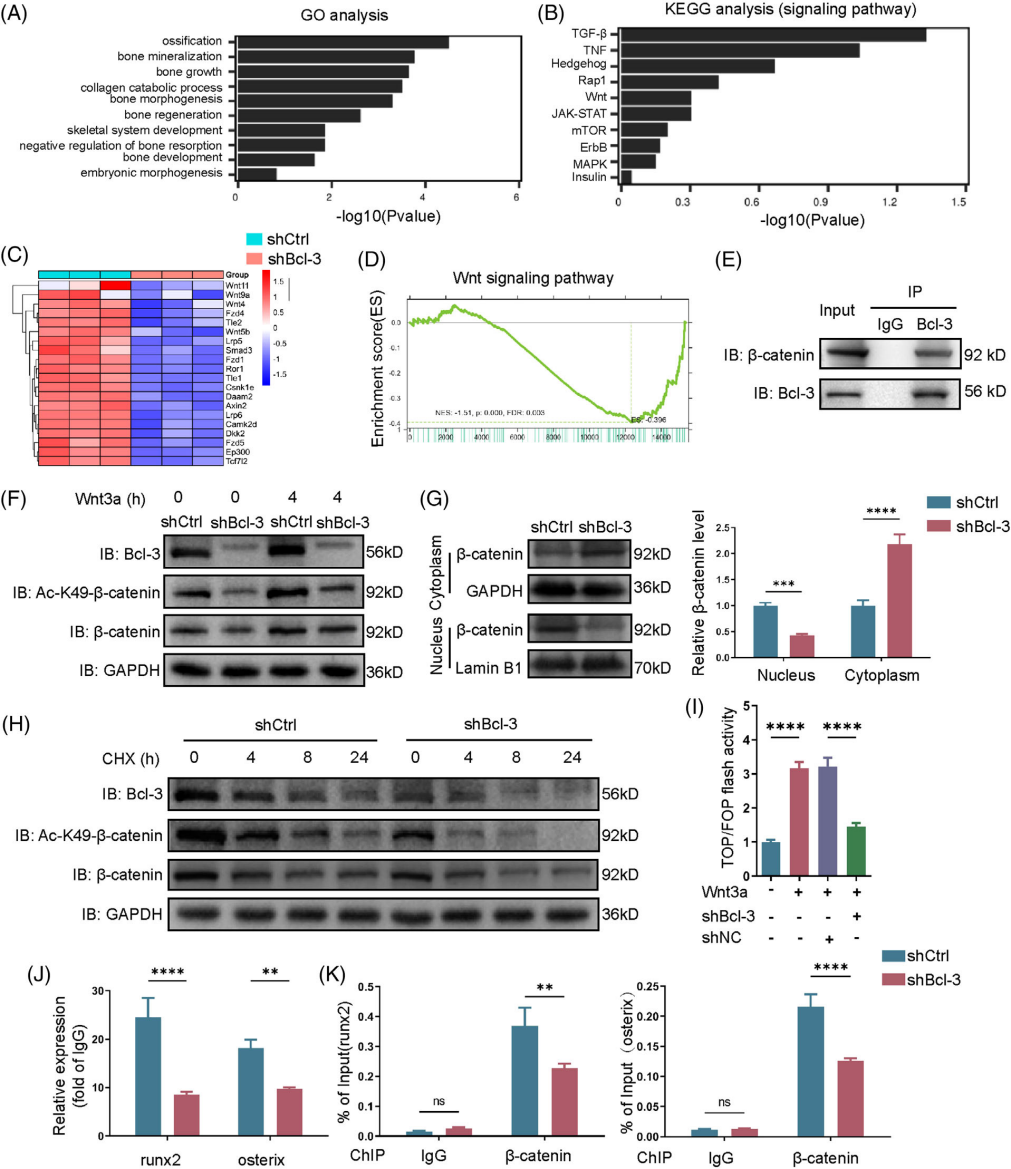
Bcl‐3 activates Wnt/β‐catenin signalling pathway and maintains β‐catenin functions in vitro. (A,B) Gene ontology (GO, Left) and Kyoto encyclopedia of genes and genomes (KEGG, Right) analysis of downregulated genes with Bcl‐3 deletion in bone marrow mesenchymal stem cells (BMSCs). (C) Heat map showed relative genes expression in Wnt pathway of shCtrl and shBcl‐3 BMSCs. (D) Gene set enrichment analysis (GSEA) showed a significant decrease of Wnt/β‐catenin gene signatures in shBcl‐3 BMSCs. (E) Lysates from BMSCs were used, and anti‐Bcl‐3 antibody was used in IP followed by immunoblot using the indicated antibodies. (F) Bcl‐3, Ac‐K49‐β‐catenin and β‐catenin were analysed by immunoblot in control and Bcl‐3‐knockdown BMSCs after treated with Wnt 3a for 0 h or 4 h. (G) The levels of Bcl‐3 in nuclear and cytoplasmic. (H) Bcl‐3, Ac‐K49‐β‐catenin and β‐catenin were analysed by immunoblot in control and Bcl‐3‐knockdown BMSCs after treated with cycloheximide (CHX, 50 mg/ml). (I) BMSCs were cotransfected with the indicated siRNA and TOP/FOP Flash reporter plasmid. (J) ChIP assays on the promoter regions of the Runx2 and Osterix genes were performed in control and Bcl‐3‐silenced BMSCs. The data are presented as the mean ± SD. **P < .01; ****P < .0001 vs. control group. Statistical analysis was performed using two‐way ANOVA. Primary antibodies: GAPDH, Abcam(ab181602); Lamin B1, Abcam(ab133741); β‐catenin, Proteintech (51067‐2‐AP); K49 acetyl‐β‐catenin, Cell Signalling Technology(9030); BCL‐3, Abcam(ab259832)

Next, we explored the molecular mechanisms of Bcl‐3 regulating β‐catenin. The Co‐immunoprecipitation (Co‐IP) analysis revealed that Bcl‐3 was bound to endogenous β‐catenin in BMSCs (Figure 3E). Depletion of Bcl‐3 leaded to decreased protein level of β‐catenin and Ac‐K49 β‐catenin after treated with Wnt 3a for 4 h (Figure 3F and Figure S5E). Moreover, nuclear translocation of β‐catenin was inhibited when Bcl‐3 was silenced (Figure 3G and Figure S5F), while Bcl‐3 overexpression in BMSCs could significantly activate β‐catenin translocation (Figure S5G). The degradation of Ac‐K49 β‐catenin was significant in Bcl‐3‐silenced BMSCs after treated with cycloheximide (CHX, Figure 3H and Figure S5H). In addition, TOP/FOP flash assays showed that Bcl‐3 depletion reduced the transcriptional activity of β‐catenin and decreased the combination of β‐catenin with promotors of *Runx2* and *Osterix* (Figure 3I–K).

Lastly, we performed OVX surgery on 8‐week‐old female mice which were intrafemorally injected with Bcl‐3 AAV2/9 or control AAV (5 × 10^12^ GC/kg) 6 weeks post‐surgery (Figure 4A). As shown in μCT, AAV2/9‐mediated Bcl‐3 overexpression prevented OVX‐induced bone loss (Figure 4B,C).

**FIGURE 4:**
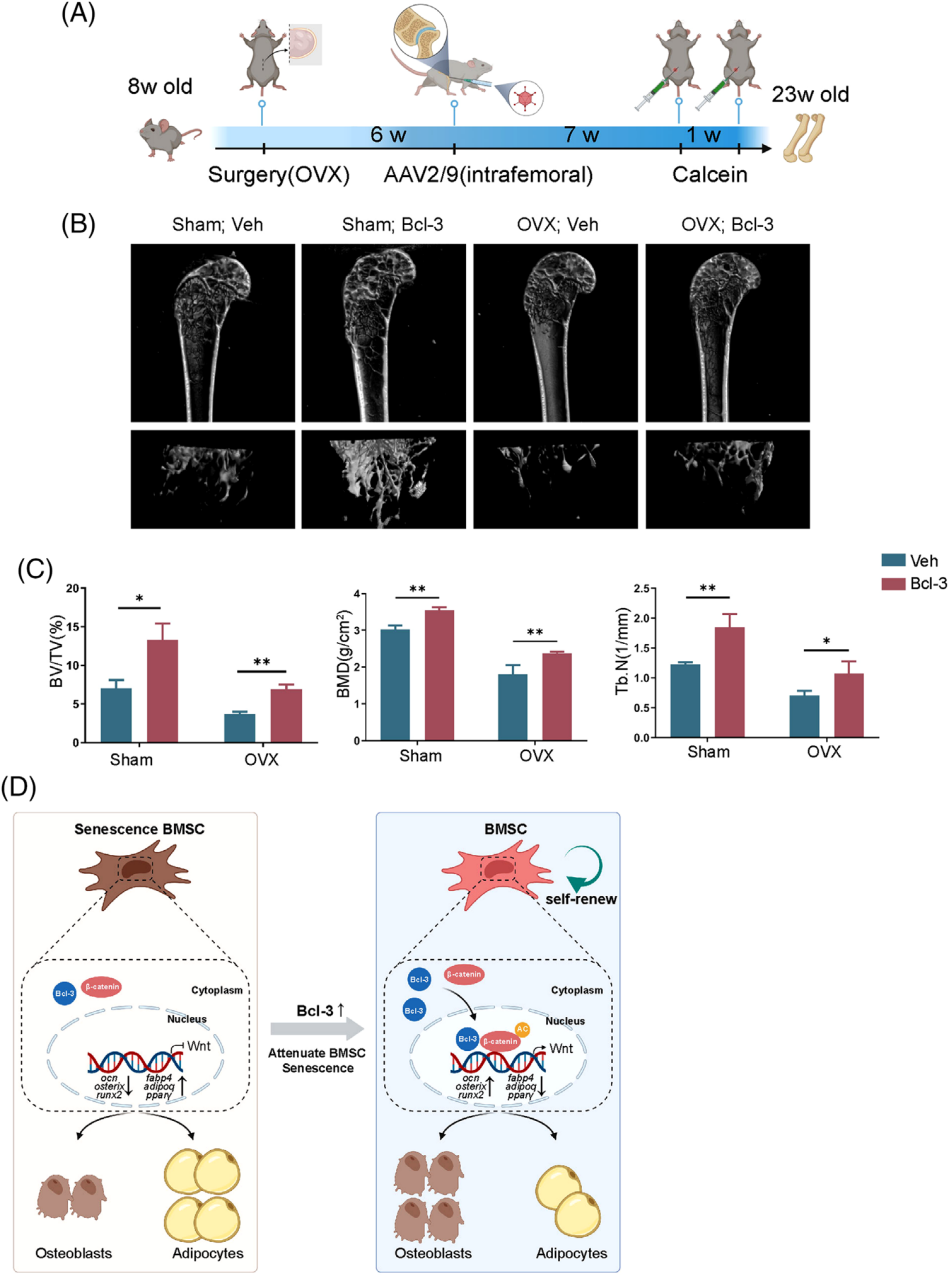
AAV2/9‐mediated Bcl‐3 overexpression prevents ovariectomy (OVX)‐induced bone loss. (A) Diagram of the study and treatment methods. (B) Representative micro‐computed tomography (μCT) images of femurs from 23‐week‐old Bcl‐3 overexpressed and WT female mice following induced OVX and sham surgery. (*N* = 3 mice/group). (C) Quantitative measurements of BV/TV, BMD and Tb.N in 23‐week‐old Bcl‐3 overexpressed and wild‐type (WT) female mice by μCT. (*N* = 3 mice/group). (D) Graphical abstract. The data are presented as the mean ± SD. *P <.05; **P <.01 vs. control group. Statistical analysis was performed using two‐way ANOVA

Osteoporosis is common among aged individuals, which could be attributed to the senescence and bone‐fat imbalance of BMSCs. During osteogenetic differentiation in BMSCs, Wnt/β‐catenin signalling functions positively. In the previous study, Bcl‐3 was proved to interact with β‐catenin and regulate Wnt signalling in colorectal tumour cells.^9^ However, the potential correlations between Bcl‐3 and Wnt /β‐catenin in osteoporosis are unclear. Bcl‐3 has been proven to activate the matrix metalloproteinase 1 expression in chondrocytes and synovial fibroblast, being the limited research on the skeletal system.^10^ Our study revealed that loss of Bcl‐3 led to bone loss, while the overexpression could be therapeutic, unveiling the substantial role of Bcl‐3 in skeletal aging. Nevertheless, the investigations into BMSCs transcriptional alterations could reflect the regulative effects of Bcl‐3 partially, calling for further explorations in the future.

Our results demonstrated that Bcl‐3 inhibited BMSCs senescence, promoted osteogenesis and decreased adipogenesis. For mechanisms, Bcl‐3 maintained Wnt/β‐catenin signalling and remained a potential target to treat age‐related osteoporosis (Figure 4D).

## CONFLICT OF INTEREST

The authors declare that there is no conflict of interest that could be perceived as prejudicing the impartiality of the research reported.

## Supporting information

Figure S1 info

Figure S2 info

Figure S3 info

Figure S4 info

Figure S5 info

**Table S1** Bcl‐3 overexpression cloning vector information and complete gene synthesis sequence
**Table S2** The primers used for PCR were as follows
