# Supplementary figures and images for: B‐cell lymphoma‐3 controls mesenchymal stem cell commitment and senescence during skeletal aging
Source: Clin Transl Med. 2022 Jul 8;12(7):e955. doi: 10.1002/ctm2.955 (PMC9270574; doi:10.1002/ctm2.955)

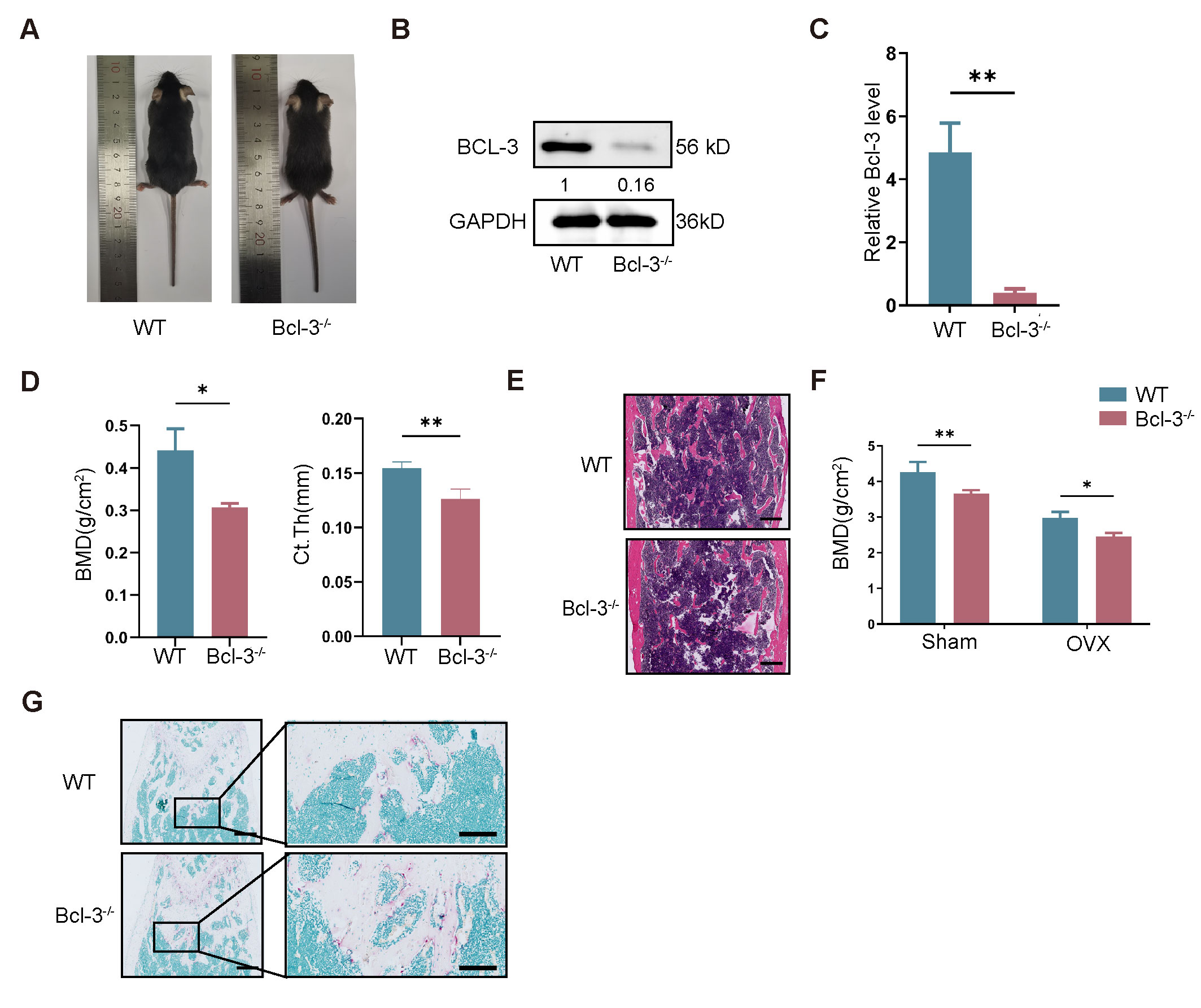

Supplement: Supplementary file 1 — Figure S1 info [file CTM2-12-e955-s005.jpg]

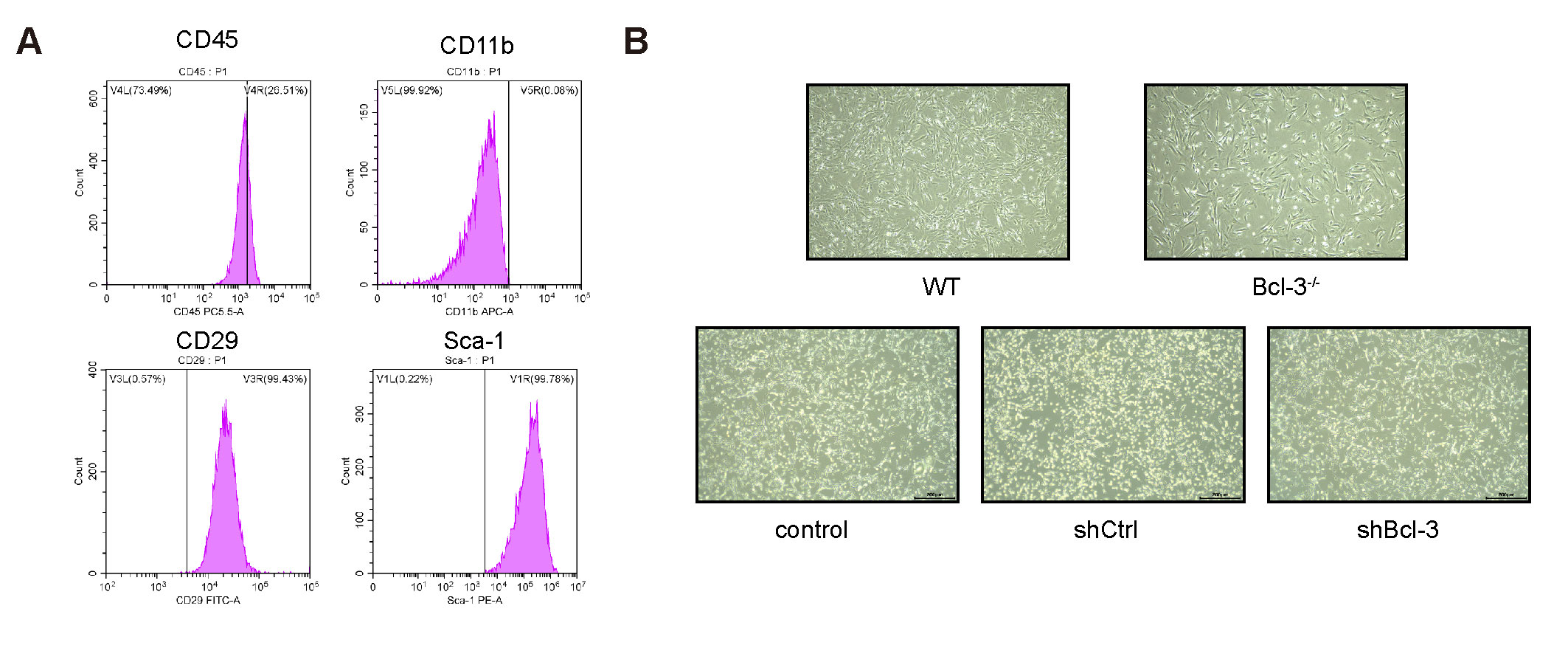

Supplement: Supplementary file 2 — Figure S2 info [file CTM2-12-e955-s003.jpg]

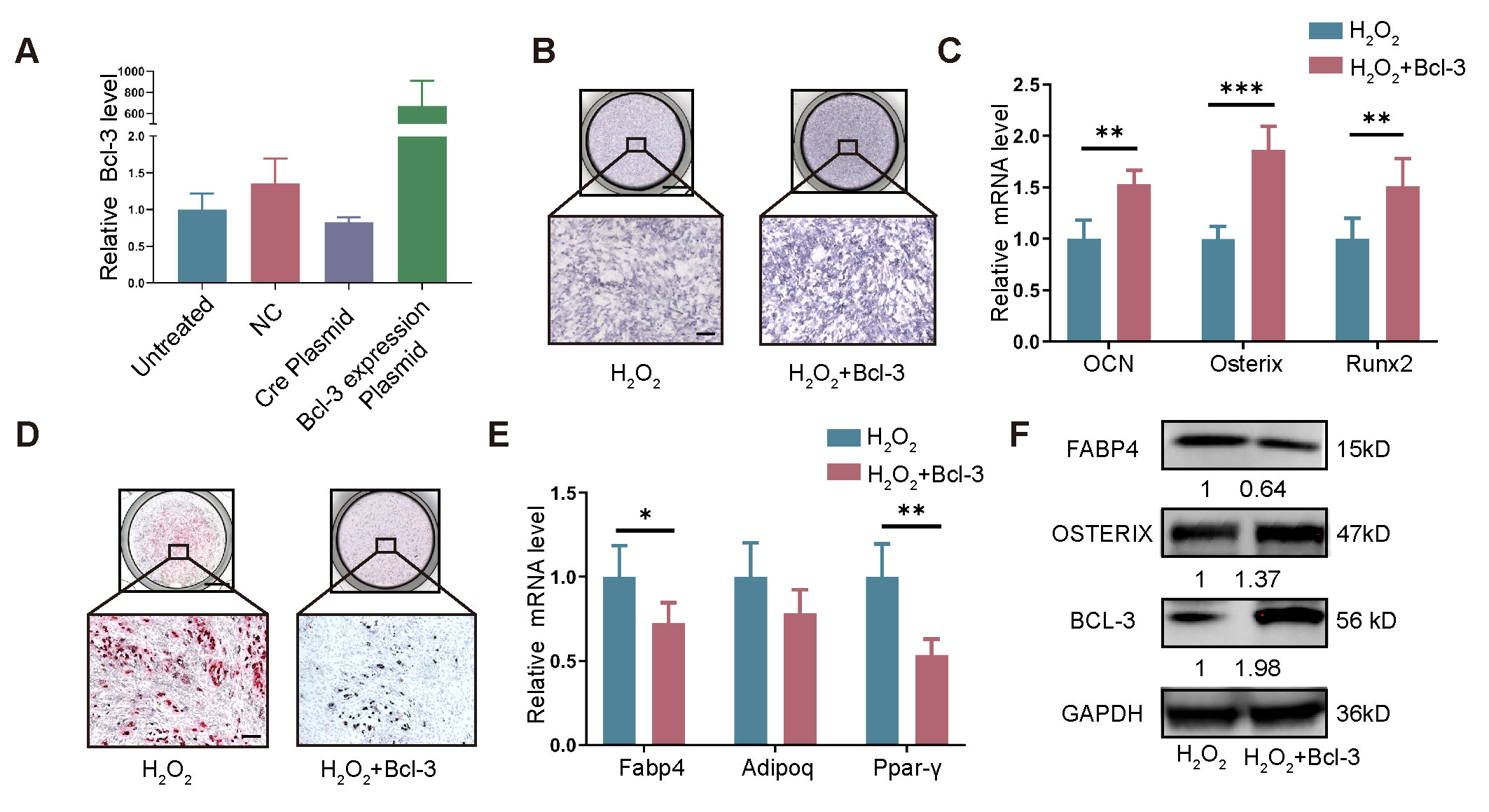

Supplement: Supplementary file 3 — Figure S3 info [file CTM2-12-e955-s001.jpg]

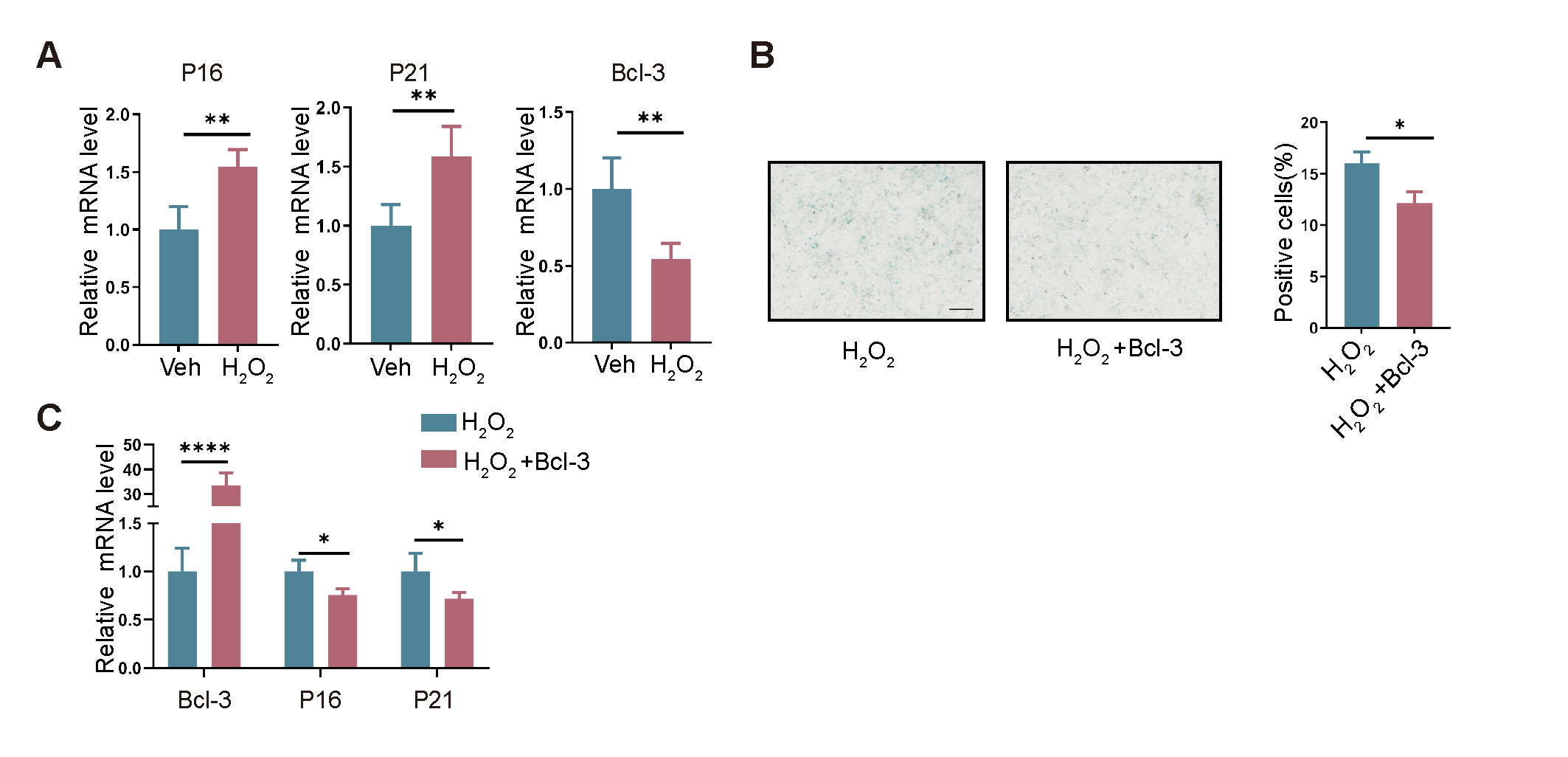

Supplement: Supplementary file 4 — Figure S4 info [file CTM2-12-e955-s006.jpg]

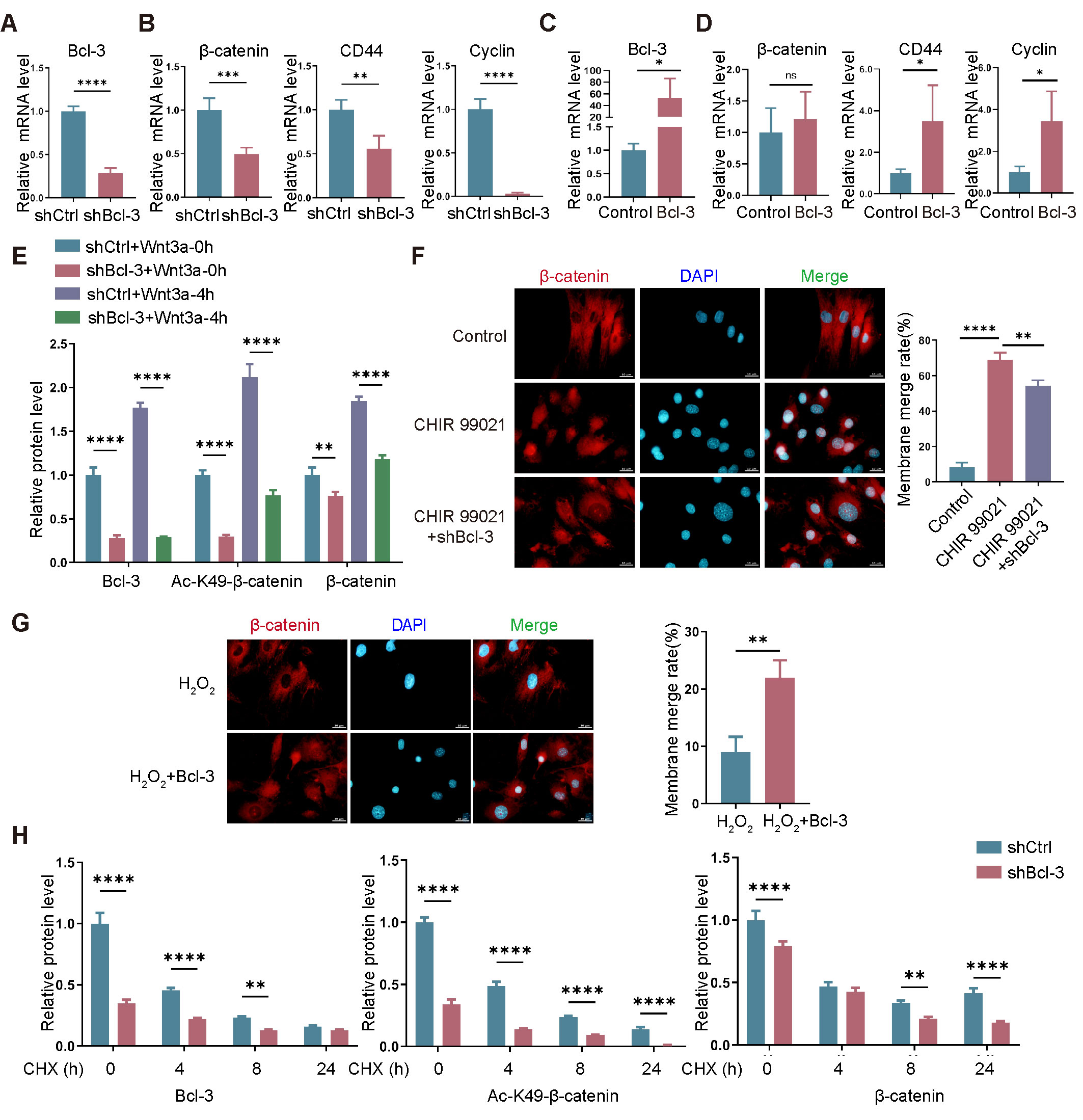

Supplement: Supplementary file 5 — Figure S5 info [file CTM2-12-e955-s002.jpg]
