# Supplementary material for: B‐cell lymphoma‐3 controls mesenchymal stem cell commitment and senescence during skeletal aging
Source: Clin Transl Med. 2022 Jul 8;12(7):e955. doi: 10.1002/ctm2.955 (PMC9270574; doi:10.1002/ctm2.955)
Supplement: Supplementary file 6 — Table S1 Bcl‐3 overexpression cloning vector information and complete gene synthesis sequence Table S2 The primers used for PCR were as follows [file CTM2-12-e955-s004.docx]

**Supplementary Table S1.** Bcl-3 overexpression cloning vector information and complete gene synthesis sequence

| Name of the carrier | PHY-026 | | |
| --- | --- | --- | --- |
| The original carrier | EF1α-MCS-CMV-zsGREEN1 | | |
| Enzyme digestion site 1 | EcoRI | Enzyme digestion site 2 | BamHI |
| The tag information | / | The label position | / |
| Vector map  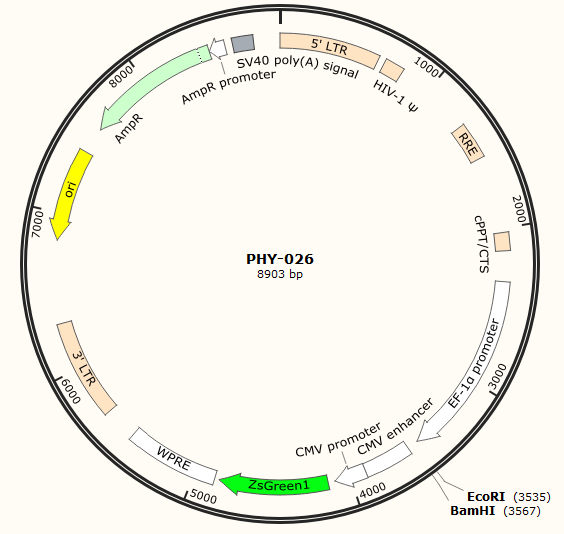 | | | |
| **Complete gene synthesis sequence**  GAATTCATAACTTCGTATAGCATACATTATACGAAGTTATCGGCAATAAAAAGACAGAATAAAACGCACGGGTGTTGGGTCGTTTGTTCAATAAAAATAAAAATAAACGGCAATAAAAAGACAGAATAAAACGCACGGGTGTTGGGTCGTTTGTTCAACTTGTTTATTGCAGCTTATAATGGTTACAAATAAAGCAATAGCATCACAAATTTCACAAATAAAGCATTTTTTTCACTGCATAACTTCGTATAGCATACATTATACGAAGTTATCGTTTAAACGCCACCATGCCCCGATGCCCCGCGGGGGCCATGGACGAGGGGCCCGTGGACCTGCGCACCCGGCCCAAGGGCACCCCGGGCGCCGCGCTGCCACTCCGCAAACGCCCGCTGCGCCCCGCGTCCCCAGAGCCCGCAACCACGCGCAGCCCCGCTGGACCCCTGGACGCCCTGCGCAGCGGCTGCGACGTCCCGGTTGTCCCTGGGCCCCCCCACTGTGTGGCCAGGCCGGAGGCCCTTTACTACCAGGGACCTTTGATGCCCATTTACTCTACCCCGACGATGGCCCCCCACTTTCCGCTGCTGAACCTGCCTACTCACCCCTACTCCATGATATGCCCCATGGAACACCCCCTTTCAGCTGATATTGCCATGGCCACTCGGGTGGATGAGGATGGAGACACGCCTCTCCACATCGCTGTGGTCCAGAATAACATAGCCGCTGTCTACCGAATACTCAGCCTTTTCAAGCTTGGGAGCCGCGAAGTAGACGTCCATAACAACCTGCGGCAGACCCCGCTCCACCTGGCTGTCATCACCACATTACCAGACATGGTCCGGCTCTTGGTGACAGCTGGTGCCAGCCCCATGGCCCTGGATCGTCACGGCCAGACTGCAATTCACCTGGCATGCGAGCACCGCAGCCCCAGCTGCCTGCAGGCCCTGCTGGACAGCGCAACCTCAGGCTCCGTGGACCTGGAGGTTCGCAATTATGAAGGGCTCACTGCCCTGCACGTGGCCGTGAACACCGGGTGCCAGGAGGCTGTTCTGCTGCTCCTGGAGCGTGGCGCGGACATCGATGCAGTGGATATCAAGAGCGGCCGCTCCCCACTCATCCACGCCGTGGAGAACAACAGCCTGAACATGGTGCAACTCCTGCTGCTGCACGGCGCCAACGTGAACGCTCAGATGTATTCTGGCAGCTCGGCTCTGCATTCTGCGTCTGGCCGCGGGCTTCTGCCTCTGGTGCGCACGCTGGTGCGCAGCGGGGCTGACAGCGGCCTCAAGAACTGTCACAATGACACACCTCTCATGGTGGCGCGCAGCCGCAGGGTCATTGATATCTTAAGGGGGAAGGCCTCTCGGGCTGCTTCAGGGTCACAGCCTGAGCCATCCCCAGACCAAAGTGCCACCAACTCCCCCGAGAGCAGCAGTCGTCTCAGCTCCAATGGCCTCCAGTCCTCGCCAAGCTCCTCACCCTCGCTGTCTCCCCCGAAAGATGCTCCTGGCTTCCCCGCGACTCCCCAAAACTTTTTCCTTCCCACAACATCTACACCTGCCTTCCTGCCCTTCCCAGGAGTCCTCCGAGGCCCTGGCCGGCCCGTACCCCCTTCCCCAGCTCCAGGAAGCAGCGTTAACTACCCCTATGACGTGCCAGACTACGCCTATCCTTACGATGTCCCCGATTATGCTTACCCATACGACGTTCCAGACTACGCATGAGGATCC | | | |

**Supplementary Table S2**

**The primers used for PCR were as follows:**

| Bcl-3 -F | CACACGCACAAATGTGGTACAC |
| --- | --- |
| Bcl-3-R | ACCACCACTGCCCATCTTATAG |
| GAPDH-F | CGGACCAATACGACCAAATCCG |
| GAPDH-R | AGCCACATCGCTCAGACACC |
| Runx2-F | GGTACTTCGTCAGCATCCTATCAG |
| Runx2-R | GCTTCCGTCAGCGTCAACAC |
| Osterix-F | ACCAGGTCCAGGCAACAC |
| Osterix-R | GCAAAGTCAGATGGGTAAGTAG |
| Fabp4-F | AAATCACCGCAGACGACA |
| Fabp4-R | CACATTCCACCACCAGCT |
| PPARγ-F | GGGATCAGCTCCGTGGATCT |
| PPARγ-R | TGCACTTTGGTACTCTTGAAGTT |
| Adipoq-F | TGTTCCTCTTAATCCTGCCCA |
| Adipoq-R | CCAACCTGCACAAGTTCCCTT |
| Cebpa-F | TGGACAAGAACAGCAACGAG |
| Cebpa-R | TCACTGGTCAACTCCAGCAC |
| Perilipin-F | GGCCTGGACGACAAAACC |
| Perilipin-R | CAGGATGGGCTCCATGAC |
| P16-F | GATGGACGTTCAGGTGGCATA |
| P16-R | GCTTAGCAATGAAACTGCGAAGT |
| P21-F | CCTGGTGATGTCCGACCTG |
| P21-R | CCATGAGCGCATCGCAATC |
| β-catenin-F | ATGGAGCCGGACAGAAAAGC |
| β-catenin-R | CTTGCCACTCAGGGAAGGA |
| CD44-F | TCGATTTGAATGTAACCTGCCG |
| CD44-R | CAGTCCGGGAGATACTGTAGC |
| Cyclin D1-F | ATGGAAGGACCCTTGAGGC |
| Cyclin D1-R | CTTCACGGCTTGCTCGTTCT |

**The antibodies used for WB were as follows:**

| Antibodies | | | |
| --- | --- | --- | --- |
| Rabbit monoclonal anti-GAPDH | Abcam | ab181602 | 1:10,000 |
| Rabbit monoclonal anti-Bcl-3 | Abcam | ab259832 | 1:2,000 |
| Rabbit monoclonal anti-Fabp4 | Abcam | ab92501 | 1:2,000 |
| Rabbit monoclonal anti-Osterix | Abcam | ab209484 | 1:2,000 |
| Rabbit polyclonal anti-Beta Catenin | Proteintech | 51067-2-AP | 1:3,000 |
| Rabbit monoclonal anti- K49 acetyl-β-catenin | Cell Signaling Technology | 9030 | 1:1,000 |
| Rabbit monoclonal anti- Lamin B1 | Abcam | ab133741 | 1:1,000 |
| DyLight® 800 | Abcam | ab201806 | 1:20,000 |

**The antibodies used for IF and IHC were as follows:**

| Antibodies | | | |
| --- | --- | --- | --- |
| Rabbit polyclonal anti-Beta Catenin | Proteintech | 51067-2-AP | 1:2,000 |
| Rabbit monoclonal anti-Fabp4 | Abcam | ab92501 | 1:10,000 |
| Rabbit polyclonal anti-Osteocalcin | Abcam | ab93876 | 1:200 |
| Goat Anti-Rabbit IgG H&L (Alexa Fluor® 488) | Abcam | ab150077 | 1:10,000 |
| Goat Anti-Rabbit IgG H&L (Alexa Fluor® 647) | Abcam | ab150079 | 1:10,000 |
